# Effectiveness of the Natural Antioxidant 2,4,4′-Trihydroxychalcone on the Oxidation of Sunflower Oil during Storage

**DOI:** 10.3390/molecules26061630

**Published:** 2021-03-15

**Authors:** Hadeil Alsufiani, Wafaa Ashour

**Affiliations:** 1 Department of Biochemistry, Faculty of Sciences, King Abdulaziz University, Jeddah 21499, Saudi Arabia; Halsufiani@kau.edu.sa; 2 Experimental Biochemistry Unit, King Fahad Medical Research Center, King Abdulaziz University, Jeddah 21499, Saudi Arabia

**Keywords:** oxidative stability, sunflower oil, 2,4,4′-trihydroxychalcone, lipid oxidation, natural antioxidants, synthetic antioxidants, butylated hydroxytolene (BHT), vegetable oils, food industries, oil rancidity

## Abstract

This study aimed to investigate the effectiveness of 2,4,4′-trihydroxychalcone as a natural antioxidant on the oxidation of sunflower oil during an 88-day storage period and to compare its strength with the synthetic antioxidant butylated hydroxytoluene (BHT). Seven groups of the sunflower oil samples were prepared: pure oil (control), oil treated with different concentrations (100, 500, and 1000 ppm) of 2,4,4′-trihydroxychalcone, and oil treated with different concentrations (100, 500, and 1000 ppm) of BHT. Specific parameters, namely, the peroxide value (PV), acid value (AV), p-anisidine value (p-AnV), thiobarbituric acid reactive substance (TBARS) value and total oxidation (TOTOX) value were used to assess the extent of the deterioration of the oil by estimating the primary and secondary oxidation products. The results showed that 2,4,4′-trihydroxychalcone effectively decreased the production of the primary and secondary oxidation products of sunflower oil during storage, as indicated by reductions in the PVs, AVs, p-AnVs, TBARS values and TOTOX values of the sunflower oil. When compared to BHT, 2,4,4′-trihydroxychalcone showed either a similar or stronger effect in inhibiting the primary and secondary oxidation products. These findings suggest that, 2,4,4′-trihydroxychalcone is a suitable natural alternative to synthetic antioxidants to improve the oxidative stability of sunflower oil.

## 1. Introduction

Lipid oxidation, which affects the color, taste, and texture of food, is the leading cause of food quality deterioration, resulting in the decreased shelf life of food [1]. Furthermore, it can generate potential toxic compounds through the action of free radicals and thus decrease the nutritional quality of food products [2]. These toxicants are thought to cause several health problems, such as malignancy, aging, and cardiovascular diseases [3,4]. As a consequence, the oxidation of oils makes them less acceptable for consumers, leading to economic losses in food industries [5].

Over the last century, the consumption of vegetable oils has increased dramatically. One of the best of these products is sunflower oil, which includes 59% of polyunsaturated fatty acids in the form of linoleic acid and 30% of monounsaturated fatty acids in the form of oleic acid [6]. Sunflower oil is particularly susceptible to lipid oxidation due to its high unsaturated fatty acid content [7,8]. It has therefore been used as a model to evaluate the ability of different plant extracts to impede peroxidation [2,9]. To solve the issues of oxidation, food industries add synthetic antioxidants such as butylated hydroxyanisole (BHA), butylated hydroxytolene (BHT), and tertbutylhydroquinone (TBHQ) to food products to block lipid oxidative degradation [10]. However, their utilization in food industries has been heavily criticized due to their toxic and carcinogenic risks to human health [11,12]. The use of these antioxidants has therefore been limited due to their adverse effects on human organs, especially the liver [13,14]. For example, restrictions have been placed on the use of TBHQ, which is considered the most effective synthetic antioxidant, in food products in Canada, Japan and Europe [15]. In addition, BHA has been excluded from a wide list of compounds that are accepted as safe (GRAS) [16]. Thus, natural antioxidants are increasingly being adopted as effective additions to prevent rancidity in various edible oils instead of synthetic antioxidants.

Natural antioxidants have gained considerable attention as a source of biologically active compounds due to their multiple health effects [17,18], and different types of antioxidants are strongly advocated to prevent food deterioration due to their safety attributes [19]. Natural antioxidant extracts have been found to have similar activity as chemically synthesized antioxidants against the oxidation of edible oils. One of these popular plants is licorice root (*Glycyrrhiza uralensis*), which is an important Chinese materia medica frequently used in clinical practice [20]. Licorice contains different types of phytochemicals, such as tripenoids, flavanones, chalcones, and coumarins and their glycosides. To date, about 42 chalcones have been isolated, identified, and categorized as nontoxic natural ingredients [21,22]. The chalcone isoliquiritigenin (2,4,4′-trihydroxychalcone) is one of the main bioactive components in licorice and has antioxidant properties, as well as estrogenic and antitumor action [20,23]. However, no study has been conducted to determine the efficacy of 2,4,4′-trihydroxychalcone in preventing lipid oxidation under storage conditions. Accordingly, the present study aimed to investigate the effectiveness of 2,4,4′-trihydroxychalcone at three different concentrations (100, 500, and 1000 ppm) in stabilizing sunflower oil during an 88-day storage period and to compare its strength with that of the synthetic antioxidant BHT under the same conditions.

## 2. Results

### 2.1. Peroxide Value

**Figure 1:**
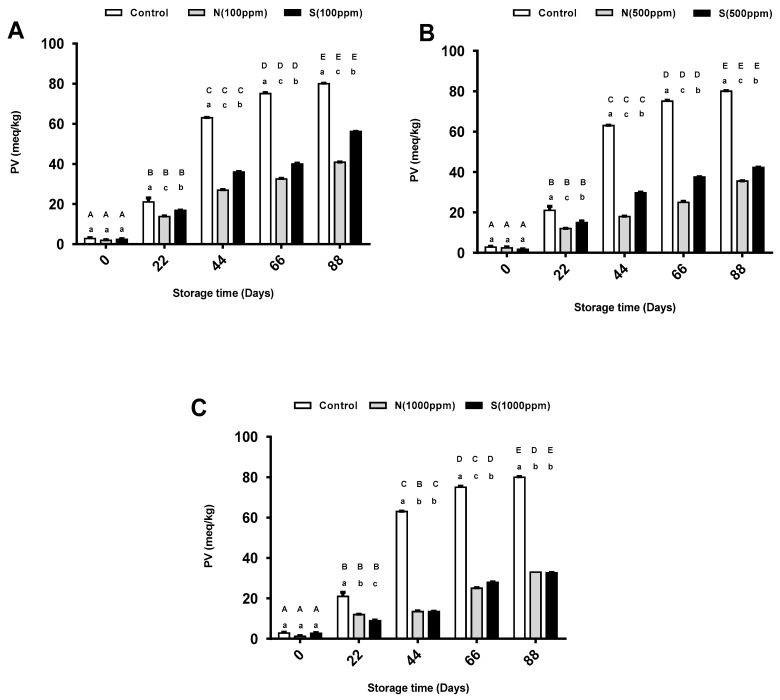
Mean peroxide value (PV) for pure sunflower oil, oil with (**A**) 100 ppm, (**B**) 500 ppm and (**C**) 1000 ppm antioxidants during storage periods (88 days). The error bars show SEM. Different upper-case letters denote a significant difference (*p* < 0.05) between PV of oil with the same dose of antioxidant but at different storage periods. Different lower-case letters denote a significant difference (*p* < 0.05) between PV of pure sunflower oil and oil with antioxidants at the same storage period. N: natural, S: synthetic.

The peroxide value (PV) method was used to measure the degree of primary oxidation of the sunflower oil for five different storage periods (i.e., 0, 22, 44, 66, and 88 days) in the presence and absence of natural and synthetic antioxidants. As the storage period increased, the PV of the pure sunflower oil increased significantly from nearly 3 meq/kg to around 80 meq/kg on day 88 (Figure 1). There were also significant increases in the PVs of the oil samples treated with both antioxidant types at different concentrations as the storage period increased (Figure 1). When comparing the PVs of the oils with the two antioxidants with that of the pure sunflower oil, the PVs of the oils with the antioxidants were significantly lower. As shown in Figure 1A,B, the highest PVs were observed for the pure sunflower oil at all storage stages, followed by the oils with 100 and 500 ppm of synthetic antioxidants, respectively. Interestingly, the oils with 100 and 500 ppm of natural antioxidant showed the lowest PVs among all the groups, a finding that was significant. Starting from day 44, the PVs of the oils with 100 and 500 ppm of natural antioxidant were nearly half and one-third those of the pure sunflower oil, respectively. When the oil was treated with 1000 ppm of antioxidants, no significant differences were observed between the two types of antioxidants in reducing the PV at any stage, except days 22 and 66 (Figure 1C). When comparing the PVs of the oils with different doses of natural antioxidant, the sunflower oil with the highest dose (1000 ppm) showed the lowest PV for all the storage periods, except days 22 and 66. Similarly, the PV of the sunflower oil with 1000 ppm of synthetic antioxidant showed the lowest PV across all storage periods when compared with the 100 and 500 ppm samples (Figure 1).

### 2.2. Acid Value

**Figure 2:**
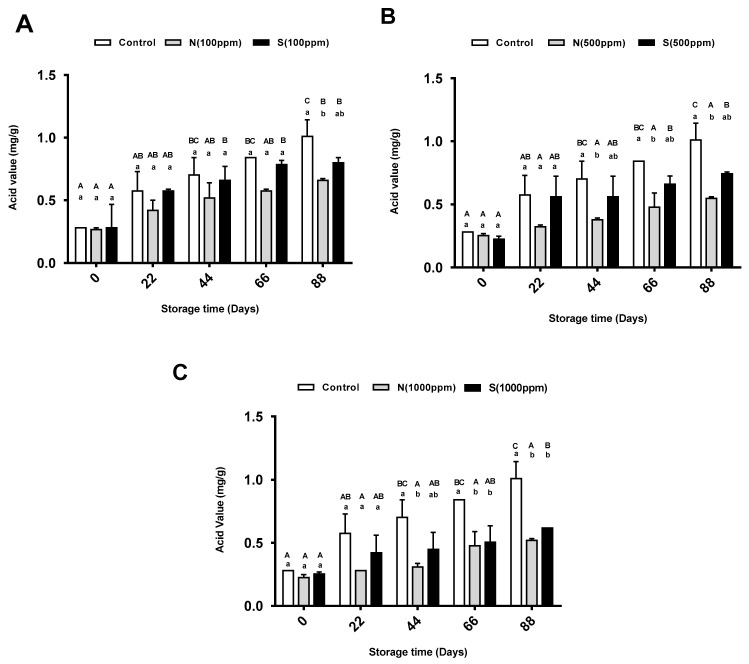
Mean acid value (AV) for pure sunflower oil, oil with (**A**) 100 ppm, (**B**) 500 ppm and (**C**) 1000 ppm antioxidants during storage periods (88 days). The error bars show SEM. Different upper-case letters denote a significant difference (*p* < 0.05) between AV of oil with the same dose of antioxidant but at different storage periods. Different lower-case letters denote a significant difference (*p* < 0.05) between AV of pure sunflower oil and oil with antioxidants at the same storage period. N: natural, S: synthetic.

The free fatty acids (FFAs) in the sunflower oil were determined by measuring the oil’s acid value (AV). Generally, as the storage period increased, the AV of the control oil sample increased; however, only the AVs at the last three storage periods (44, 66, and 88 days) were significant with zero time. Similarly, the AVs of all the treated samples increased over time. As shown in Figure 2A, the AVs did not change significantly for either the control group or the oils with 100 ppm of the synthetic and natural antioxidants between for any of the storage periods except day 88. At that time, the oil with 100 ppm of natural antioxidant showed a significant decrease in FFAs compared to the control oil. Figure 2B shows that the AVs of the oils with different treatments did not change significantly until storage day 22. After that time, the oil treated with 500 ppm of natural antioxidant showed a significant decrease in AVs compared to the control oil. The AVs of the oil treated with 1000 ppm of natural and synthetic antioxidants decreased significantly compared to the pure sunflower oil. These changes were observed after 44 and 66 days of storage for the oil with the natural and synthetic antioxidants, respectively (Figure 2C). When comparing the AVs of the oils with different concentrations of antioxidants, no significant differences were found between the AVs of the samples with the different concentrations of either antioxidant at each storage period.

### 2.3. p-Anisidine Value

**Figure 3:**
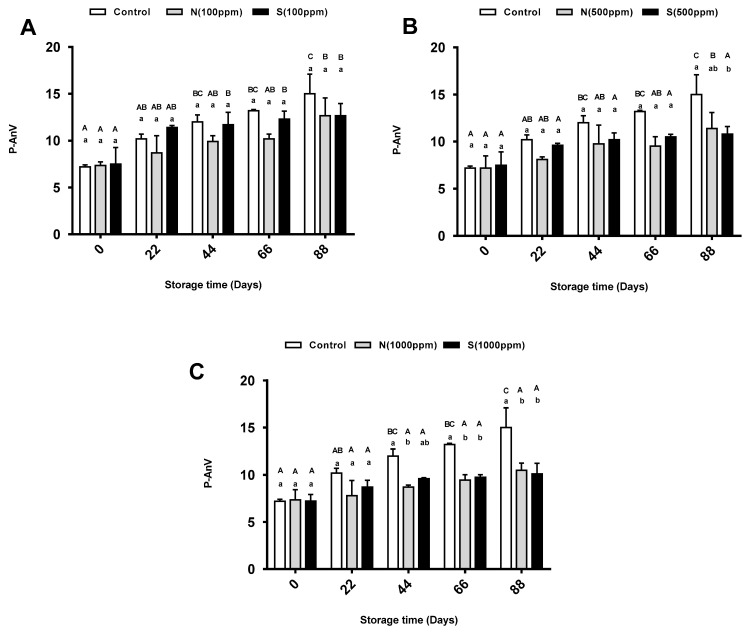
Mean p-Anisidine value (p-AnV) for pure sunflower oil, oil with (**A**) 100 ppm, (**B**) 500 ppm and (**C**) 1000 ppm antioxidants during storage periods (88 days). The error bars show SEM. Different upper-case letters denote a significant difference (*p* < 0.05) between p-AnV of oil with the same dose of antioxidant but at different storage periods. Different lower-case letters denote a significant difference (*p* < 0.05) between p-AnV of pure sunflower oil and oil with antioxidants at the same storage period. N: natural, S: synthetic.

The secondary oxidation of the sunflower oil was determined by measuring the p-anisidine values (p-AnVs). While there was a remarkably significant increase in the p-AnVs of the control samples during the storage periods, the samples with antioxidants showed a nonsignificant increase. Figure 3A shows the p-AnVs of the control oil and the oils with 100 ppm of the natural and synthetic antioxidants. The results showed no significant change between the different sunflower oil samples. Similar results were found when the oil was treated with 500 ppm of antioxidant. One exception was observed at the end of the storage time, where a significant reduction in the p-AnV of the oil with 500 ppm of synthetic antioxidant appeared compared to the control oil (Figure 3B). Increasing the dose of both antioxidants to 1000 ppm resulted in a significant reduction in the p-AnVs of the sunflower oils compared to the control oil (Figure 3C). Starting from day 44, the p-AnV of the oil treated with the natural antioxidant changed significantly compared to the control oil. Interestingly, the p-AnVs of the oil treated with the two types of antioxidant showed similar significant changes compared to the control group at 66 and 88 days of storage. When comparing the different concentrations of antioxidants, the results showed no significant changes in the p-AnVs for the samples with different concentrations (100, 500, and 1000 ppm) of the natural antioxidant, and the same results were found with the synthetic antioxidant.

### 2.4. Thiobarbituric Acid Reactive Substances (TBARS Value)

**Figure 4:**
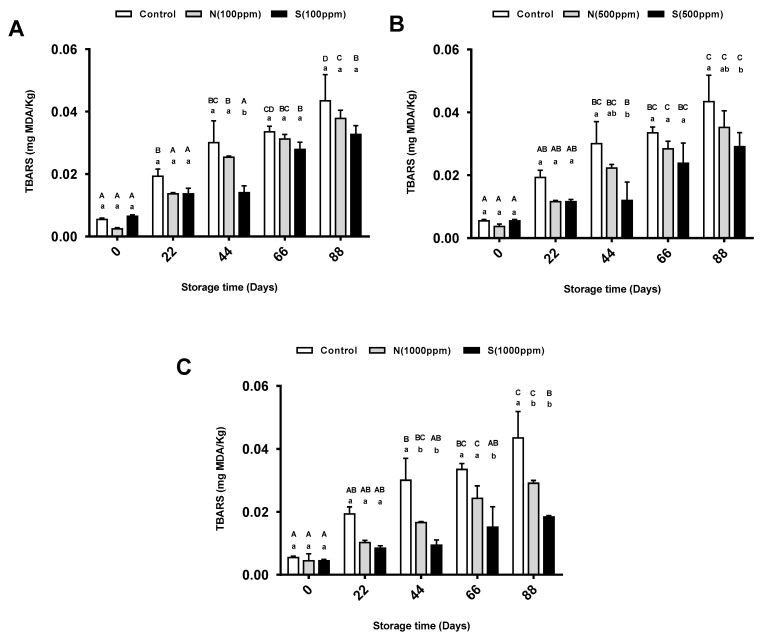
Mean thiobarbituric acid reactive substance (TBARS) value for pure sunflower oil, oil with (**A**) 100 ppm, (**B**) 500 ppm and (**C**) 1000 ppm antioxidants during storage periods (88 days). The error bars show SEM. Different upper-case letters denote a significant difference (*p* < 0.05) between TBARS of oil with the same dose of antioxidant but at different storage periods. Different lower-case letters denote a significant difference (*p* < 0.05) between TBARS of pure sunflower oil and oil with antioxidants at the same storage period. N: natural, S: synthetic.

Thiobarbituric acid reactive substance (TBARS) values were also used to determine the secondary oxidation of the sunflower oil. As the storage time increased, the TBARS values for the pure sunflower oil and the oils containing the antioxidants increased. At the end of the storage period, the highest dose of the natural antioxidant (1000 ppm) significantly decreased the formation of malondialdehyde (MDA) in the sunflower oil compared to the other two doses (100, and 500 ppm). On the other hand, the TBARS value of the oil with the highest concentration of synthetic antioxidant (1000 ppm) was significantly lower than that of the oil with the lowest concentration (100 ppm). As shown in Figure 4A, the TBARS values did not change significantly for the control group or the oil with 100 ppm of either the synthetic or natural antioxidant across any of the storage periods except day 44. At that time, the oil with 100 ppm of synthetic antioxidant showed a significant decrease in the formation of MDA in the sunflower oil compared to the control oil and the oil with 100 ppm of natural antioxidant. There was no significant effect between the different oil treatments (500 ppm) at each storage period except storage days 44 and 88. On these storage days, the TBARS values were significantly lower for the oil with 500 ppm of synthetic antioxidant compared to the pure oil, as shown in Figure 4B. When using higher antioxidant concentrations (1000 ppm), the formation of MDA was significantly lower after 44 days of storage. Both the natural and the synthetic antioxidants showed a similar effect, except at day 66 where the latter showed better results (Figure 4C).

### 2.5. Total Oxidation (TOTOX)

**Figure 5:**
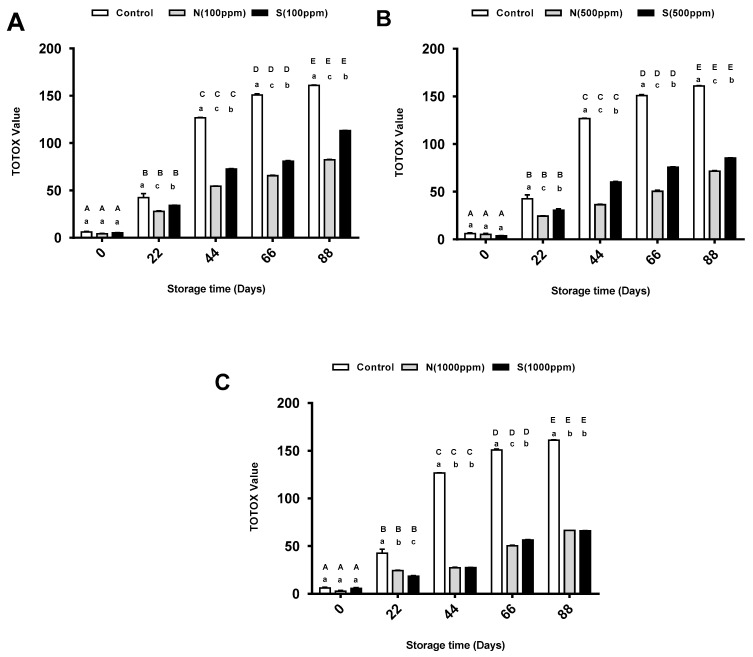
Mean total oxidation (TOTOX) value for pure sunflower oil, oil with (**A**) 100 ppm, (**B**) 500 ppm and (**C**) 1000 ppm antioxidants during storage periods (88 days). The error bars show SEM. Different upper-case letters denote a significant difference (*p* < 0.05) between TOTOX of oil with the same dose of antioxidant but at different storage periods. Different lower-case letters denote a significant difference (*p* < 0.05) between TOTOX of pure sunflower oil and oil with antioxidants at the same storage period. N: natural, S: synthetic.

(TOTOX) values represent the oxidative degradation index because they account for both primary and secondary products (i.e., peroxides and aldehydes) [24]. In this study, as the storage period increased, the TOTOX value for the pure sunflower oil significantly gradually increased significantly from less than 10 on day zero to about 160 on day 88. When comparing the TOTOX values for the oil with different doses of the natural antioxidant, the lowest values were observed for the oil with the highest dose of antioxidant (1000 ppm) across all storage periods, except days 22 and 66. At these times, the TOTOX values for the oils with 500 and 1000 ppm were almost the same. On the other hand, starting from day 22, the lowest TOTOX values were observed for the oil with the highest dose (1000 ppm) of synthetic antioxidant. Interestingly, the TOTOX values of the oil with this dose were nearly half those of the oil with the lowest dose (100 ppm) at storage days 22, 44, and 88. As shown in Figure 5A, the highest TOTOX values were observed for the pure sunflower oil at all storage stages, followed by the TOTOX value for the oil with 100 ppm of synthetic antioxidant. Interestingly, the oil with 100 ppm of natural antioxidant showed the lowest TOTOX value among all the groups, which was significant. Figure 5B shows that the TOTOX value of the oil with 500 ppm of natural antioxidant was the lowest one across all storage periods, which was significant. In addition, the TOTOX value for the control group was nearly three times that of the oil with the natural antioxidant and two times that of the oil with the synthetic antioxidant. The TOTOX values for the oils with 1000 ppm of the natural and synthetic antioxidants were significantly lower compared to that of the pure sunflower oil (Figure 5C).

## 3. Discussion

The present study evaluated the effects of 2,4,4′-trihydroxychalcone as a natural antioxidant at different concentrations (100, 500, and 1000 ppm) on the oxidative stability of sunflower oil during storage for 88 days. These effects were compared to those of pure sunflower oil and sunflower oil with BHT. The primary and secondary oxidative compounds produced during the storage periods were determined using various assays, including PV, AV, p-AnV, TBARS, and TOTOX.

### 3.1. Effects of 2,4,4′-Trihydroxychalcone and BHT on PV

PVs are used to detect peroxide formation in the early oxidation stages of lipids [25]. In this study, regular increases in the PVs as a function of storage were observed for the all the sunflower oil samples at all time intervals, and these were attributed to the formation of primary oxidation in the samples. The control samples showed remarkably significant increases in PVs compared to all the treated samples during the 88-day storage period. Such increases in PVs are due to the high content of unsaturated fatty acids of sunflower oil, which are sensitive to oxidation. As the degree of unsaturation increases, both the rate of formation and amount of primary oxidation compounds will increase and accumulate until completion of the induction duration [26]. The present results are consistent with the data reported by several researchers [27,28,29] who found similar increases in PVs although they evaluated the oxidative stability of sunflower oil under accelerated storage. In the present, the addition of natural and synthetic antioxidants to the sunflower oil at different concentrations reduced the PVs of the samples compared to the pure oil. These reductions indicated that 2,4,4′-trihydroxychalcone and BHT are effective antioxidants and stable until 88 days of storage. The results further indicated that the good antioxidant capacity was due to the higher the concentrations of antioxidants. Accordingly, the higher concentrations of the natural and synthetic antioxidants, the stronger their effectiveness. When comparing the effect of the natural antioxidant with the synthetic antioxidant, the natural antioxidant demonstrated a better effect. This better effect could have been attributable to the differences in the chemical structures of the antioxidants. 2,4,4′-trihydroxychalcone has three OH groups attached to the aromatic rings in its structure, while BHT has only one. Thus, 2,4,4′-trihydroxychalcone provides more sites for the formation of combinations with hydroperoxides, which may explain its higher activity [30].

### 3.2. Effects of 2,4,4′-Trihydroxychalcone and BHT on AV

The AV is a standard parameter used to measure the rancidity of oils and is an indicator of fat hydrolysis. In this study, an increment in the amount of FFAs as a function of storage was observed for all the sunflower oil samples at all the time intervals, which indicated the hydrolysis of triglycerides. The structure of these FFAs comprises a hydrophobic and a hydrophilic group. The hydrocarbon chain is the hydrophobe, and the carbonyl group is the hydrophilic part. The carbonyl group of these components is ideally concentrated on the surface of edible oils where it reduces the surface tension, increases the rate of oxygen diffusion from the headspace into the oil, and thus accelerates the oxidation of the oil [31]. In this study, the addition of the natural and synthetic antioxidants caused significant reductions in the FFA values of the sunflower oil during the 88-day storage period. The total increase in the FFA values during the storage periods were in the order of control > BHT > 2,4,4′-trihydroxychalcone. It was clear that the highest concentration of 2,4,4′-trihydroxychalcone (1000 ppm) was able to hinder the hydrolysis of the triglycerides as was the BHT at the same concentration. Similar results were reported by Sadeghi et al. [32], who found that the sunflower oil with the higher concentration of natural antioxidant (Ferulago angulate essential oil) showed the lowest FFA content compared to the oils with lower concentrations (250 and 125 ppm) of the antioxidant. The reducing effect of 2,4,4′-trihydroxychalcone on FFA content may be due to the donation of hydrogen atoms to free radicals and their conversion to more stable nonradical products [33]. This compound is categorized as a flavonoid with various aromatic ring substitutions, thus it can be considered a hydrogen-donating antioxidant [30]. Higher concentrations of 2,4,4′-trihydroxychalcone can therefore provide better protective activity, and can preserve sunflower oil for a long time at room temperature.

### 3.3. Effects of 2,4,4′-Trihydroxychalcone and BHT on p-AnV

The p-AnV is used to measure the products of secondary lipid oxidation produced when hydroperoxide decomposes into aldehydes, carbonyl, ketones, and carboxylic acids. This decomposition creates the rancidity flavor of oil [34]. The primary and secondary lipid products must be simultaneously detected to confirm that primary oxidation is occurring. For this reason, the p-AnVs were also analyzed in our study. The results showed a change in the p-AnVs for the control sample during the 88 days of storage, with maximum values of 18.90 ± 6.90. This change reflects the magnitude of the aldehyde formation in the oils [35,36]. The addition of p-anisidine, which contains amino groups, to the samples caused a reaction with the aldehyde carbonyl leading to the formation of a Schiff base, which absorbs light at 350 nm [37]. Although the control samples showed the highest p-AnVs, the lowest p-AnVs were for the samples with the highest concentration (1000 ppm) of both antioxidants. In a study conducted by Ling et al., the sunflower oil with natural antioxidant (unripe banana peel extracts) at the higher concentrations (200, and 300 ppm) demonstrated the lowest p-AnVs compared to the oil with a lower concentration (100 ppm) under accelerated storage. They concluded that unripe banana peel extracts are capable of retarding the oxidation of sunflower oil effectively when used at higher concentrations [29]. According to the U.S. Food and Drug Administration (2019), the maximum safety limit of BHT in food products is 200 ppm, while the Thai Food Regulations stipulate a limit of 75 mg BHT/kg for edible fats and oils (Ministry of Public Health, Thailand). Accordingly, 2,4,4′-trihydroxychalcone could be considered a good source of natural antioxidants in the food system given the maximum safety limit of BHT and the extended shelf-life of unsaturated edible oils.

### 3.4. Effects of 2,4,4′-Trihydroxychalcone and BHT on TBARS

TBARS is the most common method used to measure secondary oxidation products such as MDA, which is the standard biomarker generated from the decomposition of lipid hydroperoxide [38]. The MDA level in an oil sample can be determined through its reaction with thiobarbituric acid (TBA) to form a pink TBA–MDA complex [34]. The TBARS values in the present study for the sunflower oil samples with and without antioxidants at different concentrations (100, 500, and 1000 ppm) continued to increase from the start of storage (day zero) until the last day of storage (day 88). This graduated increase in the TBARS values indicates that primary oxidation products are being converted into secondary oxidation products [39]. These results corresponded with the p-AnVs obtained. The findings of the present study were also in accordance with the results obtained in other studies [5,29] but under accelerated storage. Both 2,4,4′-trihydroxychalcone and BHT produced lower TBARS values in the sunflower oil samples in this study. This could be explained by the chemical structure of 2,4,4′-trihydroxychalcone which contains three-OH groups that can form complexes with MDA [30,40]. Although the addition of 500 ppm of BHT was more effective than 2,4,4′-trihydroxychalcone, increasing the concentration to 1000 ppm led to a comparable inhibitory effect of both antioxidants. These results are consistent with the data reported by Chong et al. [6], who found that sunflower oil with 100 and 200 ppm of synthetic antioxidant (BHA) had the lowest TBARS value at day 6 compared with oil with a natural antioxidant (*Garcinia mangostana* Linn. peel extracts) at the same concentrations. By day 24, the sunflower oil with the mangostana peel extracts at 200 ppm and the sunflower oil with BHA exhibited comparable inhibitory effects against the secondary oxidation. They therefore suggested that mangosteen peel extracts at higher concentrations are able to prolong the shelf life of sunflower oil for a longer time.

### 3.5. Effects of 2,4,4′-Trihydroxychalcone and BHT on TOTOX

The TOTOX values were calculated to obtain overall the oxidative stability of the sunflower oil and to measure the primary and secondary oxidation products [34]. At the end of the study period, the pure sunflower oil samples showed a significant increase in TOTOX values and reached the maximum values of 160.84 ± 6.03, which were in a regular pattern, thus indicating that oxidative deterioration was occurring in the oil. The findings of this study are similar to those of the study by Chong et al. [6], who reported that the TOTOX values of pure sunflower oil increased significantly in a regular pattern during accelerated storage. In this study, all the samples treated with natural and synthetic antioxidants at three different concentrations (100, 500, and 1000 ppm) were effective in reducing the oxidative rancidity of sunflower oil. The sunflower oil stabilized with 2,4,4′-trihydroxychalcone had significantly lower TOTOX values than the sunflower oil with BHT at concentrations 100 and 500 ppm for all storage periods, but equal or lower TOTOX values at a concentration of 1000 ppm. These results showed that 2,4,4′-trihydroxychalcone is as effective as BHT or has a better effect in improving the oxidative stability of sunflower oil. The These findings are in agreement with a recent study conducted by Metzner Ungureanu et al. [41], who found that, compared to BHT, the highest concentration (800 ppm) of their natural antioxidant (blueberry byproduct extracts) was the most efficient in reducing the oxidative deterioration process of sunflower oil at a high temperature obtained by heating. Moreover, the results of the present study are in line with the findings reported in other studies [5,6,29] where the sunflower oil was treated with other types of plant extracts and showed similar or better effects than synthetic antioxidants.

## 4. Materials and Methods

### 4.1. Oil Sample Preparation

Fresh sunflower oil was obtained from a local oil press shop in Jeddah, Saudi Arabia. The oil sample was divided into seven groups: pure oil (control), oil treated with different concentrations (100, 500, and 1000 ppm) of 2,4,4′-trihydroxychalcone (natural antioxidant) and oil treated with different concentrations (100, 500 and 1000 ppm) of BHT (synthetic antioxidant). The sunflower oil samples were prepared according to the method described by Rashidch et al. [42] albeit with minor modifications. Initially, seven containers were prepared: one for the pure oil (control), three for the sunflower oil with the natural antioxidant at concentrations of 100, 500, and 1000 ppm, and the last three for oil with synthetic antioxidant at concentrations of 100, 500, and 1000 ppm. The natural and synthetic stock antioxidant solutions were prepared by dissolving each antioxidant with pyrogallol. For the 100, 500, and 1000 ppm concentrations, 0.2, 1, and 2 mg of antioxidant were added to 2 mL of pyrogallol, respectively. Then, 2 mL of the antioxidant was added to 200 mL of sunflower oil in each container. Once the oil samples had been prepared, they were stored in a dark place for 88 days. The control samples were stored under the same conditions. To monitor the lipid peroxidation process and the antioxidant activity of both the natural and synthetic antioxidants, five parameters were assessed, namely, the peroxide value (PV), Acid value (AV) content, p-anisidine value (p-AnV), Thiobarbituric acid reactive substances (TBARS), and the total oxidation value (TOTOX). All the experiments were performed at 0, 22, 44, 66, and 88 days and repeated four times.

### 4.2. Peroxide Value

The PV is one of the most widely used measurements for assessing oxidative deterioration in oils and fats. It specifically measures the concentration of the peroxides and hydro peroxides formed in the early stages of lipid oxidation [27]. The PV measures the amount of peroxide oxygen combined in a kilogram of oil (expressed as units of milliequivalents) that are able to release iodine from potassium iodide under testing [43]. The iodine is then evaluated using a standard sodium thiosulfate [43]. In this study, 5 g of an oil sample was placed in a 250 mL flask. Then, 30 mL of an acetic acid–chloroform (3:2; *v*/*v*) solution was added until the oil dissolved. A saturated potassium iodide solution (0.5 mL) was added, and the mixture was shaken by hand for 1 min. It was kept in the dark for 5 min followed by the immediate addition of deionized water (30 mL). The oil sample was then titrated against 0.1 N sodium thiosulfate (Na_2_S_2_O_3_) with constant and vigorous agitation until the yellow color disappeared. Subsequently, 1 mL of starch indicator (1%) was added, and the titration was continued until the blue color disappeared. The blank sample was analyzed under similar conditions. The volume of titrant was used to calculate the PV according to the following equation:

$$\mathrm{Peroxide}\;\mathrm{value}=\frac{\left(S-B\right)\times N\;\mathrm{thiosulphate}\;\times 1000}{\mathrm{weight}\;\mathrm{of}\;\mathrm{oil}\left(g\right)}$$

where

Peroxide value = meq peroxide per kg of sample

S = volume of titrant (mL) for sample

B = volume of titrant (mL) for blank

N = normality of Na_2_S_2_O_3_ solution (meq/mL)

### 4.3. Acid Value

The AV provides the measure of the proportion of FFAs in a substance and is used as an indicator of fat (triglycerides) hydrolysis. The AV can be defined as the milligrams of potassium hydroxide (KOH) required to neutralize the FFAs present in a 1 g sample. In this study, the AV of sunflower oil was determined using an alkali titration method according to the AOAC official method 969.17 [44] with some modifications. One gram of oil was added to a 250 mL flask. Then, 5 mL of a fat solvent mixture (ethanol and ether) and two drops of a phenolphthalein indicator were added to the oil sample. Finally, the oil samples were tittered with 0.1 M potassium hydroxide (KOH) until a permanent faint pink color appeared. The volume of KOH was then used to calculate the AV according to the following equation:

$$\mathrm{Acid}\;\mathrm{Value}\;=\;\frac{\left(A\;-B\right)\times 0.1M\;\times 56.1}{W}$$

where

A = volume of titrant (mL) for the sample

B = volume of titrant (mL) for the blank

M = molarity of KOH

W = weight of oil (g)

### 4.4. p-Anisidine Value

P-AnV analysis is a method used to measure the secondary lipid oxidation product when hydro peroxide decomposes into carbonyl, ketones, and aldehydes, a stage that leads to the rancid flavor of oil [34]. This method is based on the reaction of the p-methoxy aniline (anisidine) and aldehyde compounds, especially 2,4 dienals and 2-alkenals, as the principal metabolites of decomposition of hydro peroxide compounds (International Union of Pure and Applied Chemistry (AUPAC)). In this study, the p-AnV was determined using the IUPAC method 2.504 [45]. Two solutions were prepared: one with the reagent (p-anisidine) and the other without it. To begin, the sunflower oil sample (2 g) was first dissolved in 25 mL isooctane (solution A). The absorbance was measured at 350 nm with a spectrophotometer and using isooctane as a blank. Thereafter, 5 mL aliquot of the above mixture (solution A) was mixed with 1 mL 0.25% of p-anisidine in glacial acetic acid (*w*/*v*) (solution B). The mixture (solution B) was shaken vigorously and kept in the dark. After standing for 10 min, the absorbance was measured at 350 nm. The blank of solution B was prepared by adding 5 mL of isooctane to 1 mL of the p-anisidine solution. The p-AnV was then calculated according to the following equation:

$$p\text{-}\mathrm{Anisidine}\;\mathrm{value}\;=\;\frac{\left[25\times \left(1.2\mathrm{As}-\mathrm{Ab}\right)\right]}{m}$$

where

As = absorbance of test solution B at 350 nm

Ab = absorbance of test solution A at 350 nm

m = mass of the substance to be examined in test solution A (in grams)

### 4.5. Thiobarbituric Acid Reactive Substances

The TBARS value is defined as the quantity of MDA (in mg) present in a 1 kg of sample. The TBA value is an index of lipid oxidation and measure MDA content. This method is based on the spectrophotometric quantification of the pink complex formed from the reaction of TBA with MDA [46]. In this study, 0.1 mL of the oil sample was added to a test tube containing a mixture of 0.8 mL distilled water, 0.2 mL sodium dodecyl sulphate (8.1%, *w*/*v*), 1.5 mL 20% acetic acid (*w*/*v*; adjusted with NaOH to pH 3.5), and 1.5 mL of 0.8% 2-thiobarbituric acid solution in water (*w*/*v*), then the mixture was homogenized. The sample was heated in a water bath (100 °C) for 60 min until a pink color developed. The tube was cooled, and the sample was then centrifuged at 4300 g for 10 min. The supernatant was finally absorbed using a spectrophotometer at 532 nm. The concentration of MDA in the sample was determined by comparing the average optical density (absorbance) of the sample to a standard curve. A five-point standard curve was used to determine the unknown. First, a stock of a tetramethoxypropane (TMP) solution at a concentration of 200 μg/mL was prepared by adding 10 μL TMP (0.997 g/mL) to 50 mL distilled water. Next, from this stock solution, five aliquots of 0.5, 2.5, 5, 7.5, and 10 mL were taken and added to each volumetric flask, and the volume was completed to 10 mL by adding distilled water to prepare the five standard solutions with 10, 50, 100, 150, and 200 μg/mL concentrations, respectively. At each storage time, fresh TMP stock and standard solutions were prepared.

### 4.6. Total Oxidation Value

The TOTOX value is obtained by measuring the primary and secondary oxidation products of a sample, which reflect the initial and later stages of oil oxidation. Based on the calculated PVs and p-AnVs, the TOTOX values of the oil samples in this study were determined using the following equation [2]:

TOTOX=2PV+p-AnV

where

PV = peroxide Value

p-AnV = P-anisidine Value

### 4.7. Statistical Analysis

The collected data were analyzed using a two-way ANOVA with the different samples (control, oil with natural antioxidant, and oil with synthetic antioxidant) at the different storage periods, followed by a comparison of the different doses (100, 500, and 1000 ppm) of the natural and synthetic antioxidants individually at the different storage periods. All the statistical analyses were performed using the statistical software Prism 7 (GraphPad, San Diego, CA, USA). All the independent analyses were replicated in quadruplicate, and the results were expressed as mean ± SEM. The statistical significance levels were based on a confidence level of 95% as *p* < 0.05.

## 5. Conclusions

In the present study, different assays were used to evaluate the efficacy of the antioxidant 2,4,4′-trihydroxychalcone at 100, 500, and 1000 ppm concentrations in sunflower oil. It worked effectively to inhibit the formation of the primary and secondary oxidation products of sunflower oil during the 88 days of storage. It also showed either comparable or better effects than the synthetic antioxidant. The findings indicated that 2,4,4′-trihydroxychalcone can be used as a potent source of natural antioxidant to stabilize of sunflower oil. Future studies should focus on investigating the effects of this natural antioxidant on the oxidation of other types of edible oils.
